# Antiaging Effects of Dietary Polysaccharides: Advance and Mechanisms

**DOI:** 10.1155/2022/4362479

**Published:** 2022-07-12

**Authors:** Wei Xu, Shuai Han, Mengzhen Huang, Jiaxin Yin, Feiyan Yang, Feijun Luo

**Affiliations:** ^1^Hunan Key Laboratory of Grain-Oil Deep Process and Quality Control, Hunan Key Laboratory of Forestry Edible Resources Safety and Processing, Central South University of Forestry and Technology, Changsha, Hunan 410004, China; ^2^Hunan Food and Drug Vocational College, Department of Food Science and Engineering, Changsha, Hunan 410208, China

## Abstract

Aging is a process in which the various physiological functions of the body gradually deteriorate and eventually lead to death. During this process, the body's resistance to external stresses gradually decreases and the aging-related diseases gradually are increased. Polysaccharides are a group of active substances extracted from living organisms and are widely found in plants, animals, and microorganisms. In the last decade, a variety of natural polysaccharides from functional and medicinal foods have attracted considerable interest for their beneficial effects in the prevention of chronic diseases such as cancers, diabetes, and neurodegenerative diseases. Interestingly, these polysaccharides have also been found to delay aging by reducing oxidative damage, inhibiting telomere shortening, and being anti-inflammatory in different animal models of aging. These reviews summarized the progresses in effects of polysaccharides on antiaging and the potential mechanisms and especially focused on the signaling pathways involved in the antiaging functions. Finally, the applications and prospects of the antiaging effects of polysaccharides are discussed.

## 1. Introduction

Life expectancy has increased dramatically since the 20th century, but aging is still one of the major problems. Aging is a physiological process in which organs of body undergo an inevitable functional decline with age, a decrease in the ability of the internal environment to stabilize and cope with stress, and a gradual and irreversible deterioration of its structure and components towards death [1]. In addition to direct symptoms such as falls, reduced mobility, frailty, and incontinence, aging could also lead to metabolic-related diseases including diabetes, cardiovascular disease, cancer, and other geriatric syndromes, resulting in reduced quality of life and even death [2, 3]. In 2019, the global population aged 65 and over account for 703 million people, and the number of older people is expected to double to 1.5 billion by 2050, with the proportion of the elderly population rising further to 16% [4]. Population aging has increasingly become a more significant social issue in many countries, including China, Japan, UK, and Germany, and has attracted the attention of the international community in many areas.

Aging is a highly complex process, which also plays an important role in the normal development of the organism, the maintenance of tissue homeostasis, and the inhibition of cancer cell proliferation. It has therefore become crucial to understand how aging works, how it affects organ function, and how it can be prevented and slowed down. Over the past decades, scientific research has started with the analysis of different biological models, comparing genomic, transcriptomic, proteomic, and metabolomic analyses of long- and short-lived species, gradually leading to a partly accepted theory [5–7]. The aging process is divided into two main categories: one is physiological aging, which refers to the corresponding decline in the functions of various tissues and organs as the organism ages, resulting in an increased burden on the organism and affecting its normal life; the other is pathological aging, which refers to the presence of certain diseases that accelerate physiological aging, such as diabetes, cardiovascular disease, and cancer. These two patterns of aging are interrelated and together influence the process of aging [8]. In addition, there are many other factors that influence aging, such as smoking, alcohol consumption, radiation, and unhealthy lifestyles, and some studies suggest that psychological stress is also a major factor in aging [9]. The growing evidences demonstrate that aging is a regulated process, and theories on the mechanisms of aging include telomere shortening, accumulation of DNA damage, mitochondrial dysfunction, and epigenetic changes [10, 11].

Natural polysaccharides are carbohydrate polymeric molecules, usually linear or highly branched biomolecular compounds consisting of more than 10 homogenous or multiple monosaccharides linked by glycosidic bonds. Depending on the source, polysaccharides are classified as plant-based polysaccharides, animal-based polysaccharides, and fungal polysaccharides [12]. Studies indicate that many polysaccharides can exert various biological functions; in particular, the function of antiaging is getting more and more attention [13, 14].

## 2. Sources, Structures, and Biological Functions of Polysaccharides

Polysaccharides are found in nature from a wide range of sources and can be obtained from plants, fungi, algae, bacteria, etc. Their structures range from being linear to highly branched and include storage polysaccharides (e.g., starch and glycogen) and structural polysaccharides (e.g., cellulose and chitin) [15]. The main structures of polysaccharides in natural products are very complex and diverse, but the basic structure of their main chains usually includes a glucan, fructan, xylan, mannan, galactan, etc., or a polymer of two or more monosaccharides (e.g., galactomannan and pectin). They have a wide variety of branched structures, representing the diversity of polysaccharide structures.

Polysaccharides can be divided into two types depending on their function in living organisms. One is the nutritional polysaccharides that can store energy and nutrients, mostly branched-chain sugar-type molecules. They can be hydrolyzed by enzymes to release monosaccharides, such as starch and liver glycogen, which are soluble in hot water to form colloids. The others are insoluble in water and are structural polysaccharides, mostly straight-chain sugar-type molecules. They mainly form the supporting tissues of plants and animals, such as cellulose in plants and chitin in crustaceans. The structural classification of polysaccharides follows the analysis of proteins and nucleic acids and can be divided into primary, secondary, tertiary, and quaternary structures. As with other biological macromolecules, the advanced structure of the sugar chain is based on the primary structure, which is more complex. It is related to the relative molecular mass, the type and proportion of monosaccharides and glyoxylates, the type of monosaccharide residues, and the order of attachment between the individual monosaccharide residues [16].

Polysaccharides have a variety of biological functions. For instance, polysaccharide fraction isolated from a *Cordyceps sinensis* (CS) could stimulate iNOS expression and release NO in Raw264.7 cells [17]. *Marine microalgae* polysaccharides could inhibit DNA topoisomerases I and II and cancer cell proliferation [18]. *Astragalus* polysaccharide (APS) could improve insulin sensitivity through activation of the AMPK signal pathway in 3T3-L1 adipocytes; combined use of APS and berberine could attenuate insulin resistance in IR-HepG2 cells through modulating the gluconeogenesis signal pathway [19–21]. In this review, we summarized the efficacy and mechanisms of action of certain polysaccharides from medicinal plants, fungi, fruits, vegetables, and medicinal foods in the field of antiaging.

## 3. Antiaging Effects of Polysaccharides

### 3.1. The Theory of Aging

Telomere theory is one of the important aging theories. Telomeres are a class of DNA⁃protein complex structures that have the function of closing the ends of chromosome arms and maintaining genomic stability, which are organized into a ring-like structure called a T-loop and are associated with specific proteins, including those that make up the shelterin complex [22]. In all mammals, telomeres are formed by highly conserved hexameric (TTAGGG) tandem repeat DNA sequences [23]. The two main functions of telomeres are to protect the ends of chromosomal arms from inappropriate DNA repair mechanisms preventing the degradation of genes near the ends of chromosome arms due to incomplete DNA replication [24]. Thus, during cell division in humans and other animals, telomeres become progressively shorter in length as DNA continues to be replicated and is continuously depleted to protect chromosome ends. When telomere lengths become very short, the ability to maintain the shelterin complex is lost. In turn, the inhibition of the DNA damage response pathway by the shelterin complex is released and the cell cycle leaves the G1 phase and enters the G0 phase [25]. Upon leaving the cell cycle, the cell enters senescence or apoptosis.

Harman [26] proposed the free radical theory of aging, which states that intracellular reactive oxygen species (ROS) may be one of the most important causes of aging, and aging is caused by an increase in the production of ROS and their accumulation in the body. Under this theory, the level of ROS in the body is in a dynamic equilibrium, and the levels of oxidative stress and antioxidant defenses are in relative balance. However, with age, the secretion level of antioxidant enzymes in the body decreases and the dynamic equilibrium is imbalanced resulting in a large accumulation of ROS in the cells, which leads to the destruction of the biofilm system and impairment of normal cellular functions, thus accelerating the onset of aging. In addition, as the main site of cellular energy metabolism, oxidative phosphorylation of mitochondria is the most important pathway for ROS production. Harman further developed the theory of mitochondrial free radical senescence based on the free radical senescence theory, pointing out that mitochondrial oxidative damage and its dysfunction are the root cause of senescence [27]. Therefore, reducing the level of ROS in the body has become an effective intervention in the research on delaying aging.

The molecular mechanisms of aging and cellular senescence are an extremely complex cascade of reactions involving multiple influences. In addition to the molecular mechanisms described above, there are many other factors that can influence organismal aging. For example, DNA damage accumulation, mitochondrial dysfunction, and epigenetic regulation. In addition, activation of tumor suppressor genes such as p53 and Rb, lamin A/C (LMNA) point mutations, endocrine dysfunction, disruption of the immune system and proteostasis, dietary control, and the mammalian target of rapamycin (mTOR) signal pathway are all closely associated with the onset of aging [28]. In recent years, scientists have continued to study the mechanisms of aging in depth and have proposed various novel theoretical ideas. These theoretical perspectives have made meaningful advances in understanding the mechanisms of senescence; for example, FasL/Fas signaling could promote oocyte senescence [29], and the p53 signaling pathway mediates the human umbilical cord vascular endothelial cell senescence [30]. These theories, which explore the molecular mechanisms and signal pathways of aging from different perspectives, suggest that they are not antagonistic but complementary.

### 3.2. Animal Investigations of Antiaging Effect

*Drosophila* is a widely used invertebrate model to investigate the pathology of a variety of diseases and to examine the effects of various active ingredients, including polysaccharides. Studies on the antiaging effects of polysaccharides date back as far as 2008. A study analysed the effects of different molecular weight polysaccharides isolated from porphyran on the longevity and viability of *Drosophila melanogaster* and showed that large molecular weight (49 kDa) polysaccharides significantly prolonged the life span of *Drosophila melanogaster*. Compared to controls, porphyran polysaccharides also enhanced the mating ability of middle-aged fruit flies, while small molecular weight (8.2 kDa) polysaccharides increased survival time in heat stress tests [31].

Interestingly, the antiaging activity of polysaccharides from *Cordyceps cicadae* (CP) also appears to be correlated with molecular weight. Zhu et al. [32] evaluated the in vitro antioxidant and in vivo antiaging activities of CP extracted at different ethanol concentrations (30%-80%) and showed that CP70 (*Cordyceps* polysaccharides extracted at 70% ethanol concentration) significantly prolonged the life span of *Drosophila*. This effect was attributed to the upregulation of the expression levels of the antioxidant-related genes catalase (CAT), superoxide dismutase (SOD) 1, and MTH in *Drosophila*. Another study also confirmed the antiaging effect of polysaccharides contributing to their antioxidant capacity; for example, polysaccharides from *Chlorella pyrenoidosa* (PCPPs) are also a potential life-extending natural antioxidant [33]. PCPPs increased the total activities of endogenous antioxidant enzymes such as SOD, glutathione peroxidase (GSH-PX), and CAT in *Drosophila melanogaster*, and the average life span of male and female *Drosophila melanogaster* treated with PCPPs increased by 11.5% and 10.6%, respectively. Zhang et al. [34] found that *Sargassum fusiforme* polysaccharides (SFPS), a heteropolysaccharide extracted from the *brown alga Sargassum fusiforme*, elevated SOD, CAT, and GSH-Px activities and promote longevity by enhancing the Nrf2-mediated antioxidant pathway during the aging process in *Drosophila melanogaster*. *Premna microphylla* Turcz (pPMTLs) is a traditional Chinese medicine mainly found in the mountains of Southeastern China, and its roots, stems, and leaves can be used as medicine to detoxify, relieve swelling, and stop bleeding. A recent study found that polysaccharides from pPMTLs could extend the life span of *Drosophila* through an anti-inflammatory mechanism [35].

*Caenorhabditis elegans* (C. *elegans*) have also been used in several instances to test the antiaging activity of polysaccharides. Yuan et al. [36] suggest that two fractions of neutral polysaccharides from *Rehmannia glutinosa* (NPRRP and NPRR) could increase SOD and CAT activities in *C. elegans*, scavenging excess ROS and reducing lipofuscin expression in nematodes to prolong life span. Gu et al. [37] investigated the effects of polysaccharides from *Auricularia auricula* (AAPs-H) on locomotor behaviour, longevity, antioxidant-related enzyme activities, and antioxidant levels in *C. elegans*. AAPs-H could improve the antioxidant defense system of *C. elegans* and upregulate oxidative stress-related genes to prevent stress damage in *C. elegans* and extend its life span. Moreover, *Sophora moorcroftiana* (*S. moorcroftiana*) is a dwarf shrub of the legume family endemic to Tibet, China. A soup of its seeds has been used in Chinese folk medicine for anti-inflammation, detoxification, and infectious diseases. Zhang et al. [38] discovered that polysaccharides from *S. moorcroftiana* seeds significantly improved the survival rate of *C. elegans* in acute heat stress, prolonged the life span of *C. elegans*, and improved its reproduction.

In a rat model, Zheng [39] found that injection of *Polygonatum sibiricum* (PS) polysaccharides significantly increased the SOD and GSH-Px activities of renal tissues in the D-galactosamine- (D-gal-) induced aging rats. The decrease in malondialdehyde (MDA) content and *β*-galactosidase activity indicated the antiaging effect of PS polysaccharide. Cysteinyl aspartate-specific proteinase-3 (caspase-3) is the most critical apoptosis-executing protease in the apoptotic process, which cleaves Bcl-2 protein and triggers apoptosis upon activation. Moreover, the activity of Bax and caspase-3 in the cells of senescent mice was increased. Jing et al. [40] found that the expression level of Bcl-2 was increased and the expression levels of Bax and caspase-3 were decreased in the cells of senescent mice gavaged with polysaccharides from *Athyrium multidentatum* (Doll.) Ching (AMC) rhizome, indicating that *Athyrium multidentatum* polysaccharide could inhibit cell apoptosis and resist senescence. In addition, oral administration of polysaccharides of *Fructus corni* (PFC) significantly restored the protein levels of Bcl-2 and reduced the expression of Bax and caspase-3 in ovarian granulosa cells of senescent mice. It suggests that PFC can prevent the apoptosis of granulosa cells in the ovary of aging mice, thus reducing the aging rate of the cells [41]. *Dendrobium* is one of the most important herbal foods for the treatment of geriatric diseases, and *Dendrobium officinale* polysaccharides (DOP) could reduce cognitive impairment in accelerated aging mouse models by activating microglia [42].

### 3.3. Cell Investigations of Antiaging Effect

In terms of cellular studies, Tong et al. [43] investigated the effects of sulfated *Bupleurum chinense* polysaccharides (BCPs) on hydrogen peroxide-induced free radical scavenging activity in mouse lung endothelial cells and their effects on oxidative stress-induced senescence. The results showed that these polysaccharides play an important role in free radical scavenging and are resistant to oxidative damage, and they may be considered novel pharmaceutical products with potential antioxidant and antiaging effects. *Angelica sinensis* polysaccharides (ASP) could inhibit vascular endothelial cell senescence by enhancing protein kinase B (AKT)/hTERT phosphorylation and inhibiting oxidative stress [44]. They can also delay senescence in response to X-ray-induced hematopoietic stem cells in mice by inhibiting oxidative damage and downregulating p16 mRNA expression [45]. Furthermore, *Lycium barbarum* polysaccharides (LBP) protect against oxidative stress-induced apoptosis and senescence in human lens epithelial cells [46]. They could suppress zebrafish embryonic cell apoptosis and senescence through a p53-mediated pathway [47] and delay human umbilical vein endothelial cell senescence by downregulating the expression of p53 and p15 [48].

### 3.4. Clinical Investigations of Antiaging Effect

In terms of clinical research, LBP has pioneered antiaging clinical studies. Amagase and Nance [49] conducted a randomized, double-blind, placebo-controlled clinical study using standardized LBP juice (containing 13.6 mg/mL LBP) given to healthy adults for 14 days. The results demonstrated that daily administration of LBP for 14 days increased subjective perceptions of overall well-being and improved neurological/psychological performance and gastrointestinal function in the subjects. Amagase et al. [50] further conducted a randomized, double-blind, placebo-controlled clinical study in which 50 healthy Chinese adults aged 55-72 years were recruited and treated with LBP juice containing 13.6 mg/mL LBP at a dose of 120 mL/day or placebo (*n* = 25 per group). The results showed that LBP juice significantly increased serum SOD levels by 8.4%, GPx levels by 9.9%, and MDA by 8.7% in the subjects. These data suggest that LBP is well tolerated in humans and can promote antioxidant capacity in humans through upregulation of antioxidant enzymes.

Overall, the antiaging activity of polysaccharides warrants further exploration through a summary of cellular, animal, and clinical trials. The antiaging effects of natural product polysaccharides are listed in Table 1.

## 4. Antiaging Mechanisms of Polysaccharides

### 4.1. Regulation of Telomeres and Telomerase

In recent years, an increasing number of studies have shown that polysaccharides could exert unique antiaging effects by increasing telomerase activity or inhibiting telomere shortening. For example, *Cynomorium songaricum* polysaccharide (CSP) could exert antiaging effects by increasing telomere length in aging mice [51]. Turmeric polysaccharides could upregulate the function of telomerase reverse transcriptase, which promoted cellular immune response, tissue repair, and longevity [52]. ASPs were able to increase telomere length and enhance telomerase activity in hematopoietic stem cells of mice in a senescence model [53, 54]. However, paradoxically, ASPs have also been reported to damage telomeric regions or inhibit telomerase activity to exert anticancer effects [53]. Interestingly, reducing high telomerase activity in cancer cells may actually help to delay organism aging and related diseases [55]. In clinical studies, dietary supplementation with the polysaccharide extract of *Astragalus* enhanced telomere length in lymphocytes from healthy volunteers [56]. In recent years, the medical community has devoted significant attention to telomeres and telomerase. With the rapid development of molecular biology, more and more phytochemicals with antiaging functions by controlling telomere length and telomerase activity will continue to be discovered and fully explored.

### 4.2. Free Radical Scavenging

Oxidative stress caused by elevated levels of intracellular ROS is a major trigger for the aging process and age-related pathogenesis, yet intracellular ROS levels and oxidative stress could be reduced by plant or fungal polysaccharides [57]. Polysaccharides from *Angelica sinensis* not only protect neuronal cells from hydrogen peroxide-induced cytotoxicity but also reduce apoptosis and intracellular reactive oxygen levels. In a rat model of localized cerebral ischemia, ASP could enhance the antioxidant activity of cortical neurons, increase the number of microvessels, and improve postischemic blood flow [58]. In addition, ASP could protect mice from neural stem cell senescence by increasing the activity of SOD and T-AOC (total antioxidant capacity) and reducing the content of MDA, providing a theoretical basis for the treatment of brain aging-related diseases with polysaccharides [59]. Polysaccharide from *Trichosanthes* peel could increase SOD, CAT, and GSH-PX activities and reduce MDA levels in the liver, brain, kidney, and serum in D-galactose-induced senescent mice [60]. Besides, *Auricularia* polysaccharides scavenged free radicals and upregulated SOD levels in *Cryptorchidium hidradenum*. *Dictyophora* polysaccharides also have antioxidant and neuroprotective functions in *Cryptorchis* spp. Heat stress is an endogenous oxidative stress in cells, which can promote the production of large amounts of free radicals causing oxidative damage in the body. Polysaccharides from AMC rhizomes could reduce D-galactose-induced oxidative stress and apoptosis by activating the phosphatidyl inositol 3-kinase (PI3K)/AKT pathway, which may partially contribute to its antiaging activity [40]. Zhao et al. [31] extracted polysaccharides from *Porphyra haitanensis* and added them to the diet of *Drosophila melanogaster*, which not only increased their resistance to heat stress by 12.37% but also significantly extended the life span of male and female *Drosophila* under stress-free conditions by 8.6% and 9.34%, respectively. Taken together, polysaccharides play important roles in inhibiting the development of aging-related diseases through antioxidative stress mitigation.

### 4.3. Enhancement of Immunity

Polysaccharides play an important role in immune regulation and can regulate the immune response by modulating the expression levels of cytokines, thus achieving antiaging effects [61]. Studies showed that LBP could enhance the response of T helper 1 cells (Th1) and T helper 2 cells (Th2) in dendritic cells, which could improve immunity of the body [62]. LBP could significantly increase the lysozyme content in silkworm, which eliminated pathogenic microorganisms by forming soluble glycopeptides in the cell walls of bacteria, thus improving the body's resistance to pathogenic microorganisms and enhancing immunity [63]. *Millettia pulchra* polysaccharides (MPP) could reduce the production of proinflammatory factors such as interleukin-2 (IL-2) and interleukin-6 (IL-6) in the serum of aging mice, increased the spleen and kidney index of mice, and enhanced their immunity, thereby achieving antiaging effects [64].

### 4.4. Others

In addition, plant polysaccharides inhibit neurotoxicity and prolong the healthy life span of organisms by regulating proteostasis and alleviating or removing misfolded and abnormally aggregated proteins such as *β*-amyloid (A*β*), polyQ, or *α*-synuclein associated with neurodegenerative diseases [65]. For example, PS polysaccharides protect Alzheimer's disease neuronal cells by inhibiting neurotoxicity, and APS block polyQ40 expression, slowing down the abnormal accumulation of polyQ40 causing neurotoxicity and prolonging the life span of *C. elegans*. Fungal polysaccharides could also regulate some signal pathways closely related to life span, such as IIS, mitogen-activated protein kinases (MAPK), and mTOR pathways, to delay aging. *Ganoderma lucidum* polysaccharides (GLPs) could activate the expression of DAF-16, a life span-associated transcription factor in the IIS pathway, through the MAPK pathway to prolong the life span of *E. hidradiata* [66]. Moreover, plant or fungal polysaccharides could extend the life span of model animals or have better effects in reducing the onset of diseases associated with aging (*Drosophila*, *Hidradenia*, and rats), but their mechanisms of action are unclear. Therefore, it is an urgent scientific issue to explain how polysaccharides regulate life span extension.

## 5. Polysaccharides and Aging-Related Signal Pathways

### 5.1. Regulation of Sirt-1

Sirtuin (Sirt) was firstly discovered in nature in the 1970s. Sirts are an evolutionarily conserved family of class III histone deacetylases that depend on nicotinamide adenine dinucleotides. In mammals, there are seven species of Sirts (Sirt1 to Sirt7), which are involved in maintaining genomic stability, regulating energy metabolism, and modulating cancer stem cells [67]. Despite the fact that functions of the seven Sirts are different, they all share a conserved region for NAD+ binding and catalysis and have two structural domains, a large one consisting mainly of Rossmann folds and a small one consisting of a zinc band structure and a helix. They targeted histones and transcription factors and regulated cellular phenotypes via epigenetic modification. Sirt3, Sirt4, and Sirt5 are found in the mitochondria and could moderate oxidative stress in the mitochondria, while Sirt2 could shuttle between the nucleus and the cytoplasm [68]. Sirt1 is currently the most popular and widely studied protein in aging, and a series of studies have shown that Sirt1 could slow down cellular aging by inhibiting apoptosis, regulating metabolism, and suppressing inflammation [69, 70]. It is also involved in many age-related processes and disorders, such as neurodegenerative and cardiovascular diseases [71].

Polysaccharides could exert unique antiaging effects by activating Sirt1. Probiotics and prebiotics used to prevent and alleviate degenerative changes associated with aging have received much attention. Wang et al. [72] used *Lactobacillus plantarum* 69-2 in combination with galacto-oligosaccharide (GOS) in a mouse model of aging to assess the effects on aging. The results showed that *Lactobacillus plantarum* 69-2 and GOS could restore hepatic antioxidant activity and alleviate aging by activating the hepatic adenosine monophosphate-activated protein kinase (AMPK)/Sirt1 signal pathway. For instance, bitter melon polysaccharide could activate Sirt1-mediated *β*-linked protein deacetylation to exert neuroprotective effects [73, 74]. Lentinan (LEN) has various biological properties such as anticancer, antibacterial, antiviral, and antioxidant effects and could protect cardiomyocytes from hypoxic damage by regulating microRNA-22/Sirt1 [75]. LBP could inhibit cell death by activating Sirt1 [76, 77], and they also could exert protective effects by increasing cell proliferation, inhibiting apoptosis, and regulating Sirt1/hypoxia-inducible factor 1*α* (HIF-1*α*) pathway [78]. *Tremella* polysaccharides could activate Sirt1 and inhibit *P. aeruginosa* lipopolysaccharide-induced ROS production, apoptosis, and autophagy [79]. APS ameliorates mitochondrial dysfunction through the Sirt1 pathway and has a restorative effect on mitochondria [80]. It also attenuates endoplasmic reticulum stress and apoptosis by regulating the Sirt1-PPAR*γ* coactivator 1*α* (PGC-1*α*)/peroxisome proliferator-activated receptor (PPAR) *α*-FGF21 signaling pathway in the rat liver and the miR-204/Sirt1 axis in retinal pigment epithelial cells [81, 82]. Moreover, polysaccharides from *okra* (*Abelmoschus esculentus* (L.) Moench) could inhibit apoptosis and oxidative stress by activating the Sirt1 axis [83], while *Tremella fuciformis* polysaccharide (TFPs) could inhibit hydrogen peroxide-induced damage through upregulation of Sirt1 in human skin fibroblasts [84]. The tubers of *Apios americana* Medikus are of high nutritional value and have long been used as food in many countries. However, a study by Chu et al. [85] showed that tuber polysaccharides of *Apios americana* Medikus with an average molecular weight of 12.16 kDa significantly inhibited the lipopolysaccharide- (LPS-) induced release of nitric oxide (NO) and inflammatory cytokines from Raw264.7 cells by modulating Sirt1, as well as oxidative stress and mitochondrial dysfunction.

### 5.2. Regulation of mTOR

The mTOR pathway is a class of intracellular serine/threonine protein kinases that act as key regulatory molecules in cellular physiopathological processes, receiving, integrating, and then responding to various stimuli (hormones, growth factors, nutrition, energy, hypoxia, and stress) from both inside and outside the cell. Its mediated signaling pathways play an extremely important role in life processes [86]. Two different complexes of intracellular mTOR are mTORC1 and mTORC2. mTORC1 responds to amino acids, stress, oxygen, energy, and growth factors and is sensitive to rapamycin [87]. It promotes cell growth by inducing and inhibiting anabolic and catabolic processes and also promotes cell cycle progression. mTORC2 responds to growth factors, regulates cell survival and metabolism and the cytoskeleton, and is not sensitive to rapamycin [88]. There has been widespread interest in recent years in the study of the effects of mTORC1 on senescence. It inhibits cellular autophagy and promotes protein synthesis through growth factor and nutrient activation. Over time, this may promote cellular stress (protein aggregation, organelle dysfunction, and oxidative stress) and may lead to the accumulation of damage and reduced cellular function, thereby contributing to the development of aging-related diseases [89, 90]. Studies have also shown that inhibition of the mTOR signal pathway through gene knockout, treatment with rapamycin, or dietary restriction can delay aging in various biological models such as yeast, worms, fruit flies, and mice [91]. Dietary restriction and modulation of the cellular autophagic response are beneficial in extending life span, in which the mTOR signal pathway plays an important role [92].

GLPs are bioactive substances with antioxidant, anticancer, and neuroprotective effects [93, 94]. Liang et al. [95] showed that GLPS exerted protective effects against palmitic acid-induced apoptosis and autophagy by regulating the mTOR signaling pathway. Besides, APS enhanced autophagy by inhibiting the PI3K/AKT/mTOR pathway [96], and a sulfated dextran from cinnamon could reduce the viability of lung cancer cells by inhibiting mTOR activity [97]. Pectic bee pollen polysaccharide (RBPP-P) was isolated from *Rosa rosea* to reduce hepatic steatosis and insulin resistance by promoting autophagy through the AMPK/mTOR-mediated signaling pathway [98]. Fucoidan, a polysaccharide derived from brown seaweed, has long been used as an ingredient in some dietary supplement products, and it inhibited the PI3K-AKT-mTOR pathway [99]. In addition, *Pleurotus ostreatus* polysaccharides (POPs) inhibit the AMPK/PI3K/mTOR pathway [100]. Chitosan, an animal polysaccharide derived from the shell of crustaceans, has also been reported to inhibit the mTOR pathway [101].

### 5.3. Regulation of AMPK

AMPK is a highly conserved regulator of cellular energy metabolism. When the intracellular AMP/ATP ratio is increased, AMPK phosphorylates and activates a large number of downstream target molecules so that ATP utilization is reduced and ATP production is increased, thereby increasing cellular catabolism. When the AMP/ATP ratio is decreased, AMPK activity is inhibited, resulting in an increase in cellular anabolism [102]. AMPK is a heterotrimeric complex composed of catalytic subunits (*α*1, *α*2, *β*1, and *β*2) and regulatory subunits (*γ*1, *γ*2, and *γ*3). Expressed in various metabolically relevant organs, the different isomers of *α*, *β*, and *γ* could form various possible combinations [103]. The aging process of life is closely related to the metabolic rate, and the aging process of organisms is accompanied by a gradual downregulation of AMPK activity, which tends to be higher in young animals than in older ones. Decreased AMPK activity may be associated with numerous aging-related diseases such as cardiovascular disorders and metabolic syndrome, and activation of AMPK could extend the life span of organisms [104].

APS, an extract of *Astragalus membranaceus*, is an important bioactive component, and APS improves insulin sensitivity in mice by activating AMPK [28]. In a rat model, APS increased hepatic glycogen synthesis via activation of AMPK in a T2DM rat model [105]. Moreover, in cellular studies, APS significantly enhanced the AMPK/Sirt1 pathway in porcine alveolar macrophages to alleviate apoptosis and proinflammatory cytokine expression [106]. Palmitate-treated Raw264.7 cells produced an inflammatory response, while APS showed anti-inflammatory effects by activating AMPK that effectively ameliorated the palmitate-induced proinflammatory response [107]. *Okra* is an annual herb of the mallow family, and *okra* polysaccharides could inhibit apoptosis and oxidative stress by activating the AMPK-Sirt1-PGC-1*α* signaling axis [83]. *Epiphyllum* is the roots of a buttercup plant *Aconitum carmichaelii* Debx, and *Epiphyllum* polysaccharides protect H9c2 cells from starvation-induced cytotoxicity by activating the AMPK pathway [108]. *Schisandra chinensis* is a liver-protective herb that has been used in China for centuries. Polysaccharides are one of its main active ingredients and have been reported to improve alcohol-induced liver injury [109]. Moreover, the protective activity of *Schisandra chinensis* polysaccharides (SCPs) in mice with acute liver injury may be related to the antioxidant, anti-inflammatory, and antiapoptotic properties resulting from activation of the AMPK pathway [110]. Tan et al. studied the antiobesity effects of the polysaccharide-rich *red alga Gelidium amansii*, a hot water extract of which contained 68.54% water-soluble indigestible carbohydrate polymers. In addition, pectin is a complex polysaccharide found in plant cell walls and consists of four main types of polysaccharides: homogalacturonan, xylogalacturonan, and rhamnogalacturonans I and II, all of which could be degraded into various pectic oligosaccharides (POS). POS are considered to be new potential functional foods with a variety of health-promoting properties, and POS are thought to have a role in regulating AMPK [111]. The aging process is accompanied by a decrease in physical endurance, and polysaccharides from *Irpex lacteus*, a white-rot tamarin fungus, could increase the antifatigue activity of Kunming mice, whose ability to improve endurance is mainly achieved by activating AMPK [112]. Low molecular weight rockweed polysaccharide (LMWF), a sulphated polysaccharide derived from brown seaweed with strong anti-inflammatory and antioxidant activities, prevented nonalcoholic fatty liver disease (NAFLD) in db/db mice by activating the Sirt1/AMPK/PGC1*α* signaling pathway [113], while fucoidan polysaccharides ameliorate renal cell senescence by inhibiting autophagy-related activation of the AMPK-ULK1 signaling pathway through VE-like effects in a D-gal-induced senescence model of human proximal renal tubular epithelial cells. Wu et al. [114] extracted chicory polysaccharides (CPs) from chicory roots and fractionated it to isolate two novel polysaccharide fractions, CPs-1 and CPs-2, of which CPs-1 is a heteropolysaccharide composed mainly of sorbitan, glucose, fructose, and glucitol. CPs-1 significantly attenuated high-fat diet-induced nonalcoholic fatty liver disease in rats through AMPK activation [114].

### 5.4. Regulation of p53

**Figure 1:**
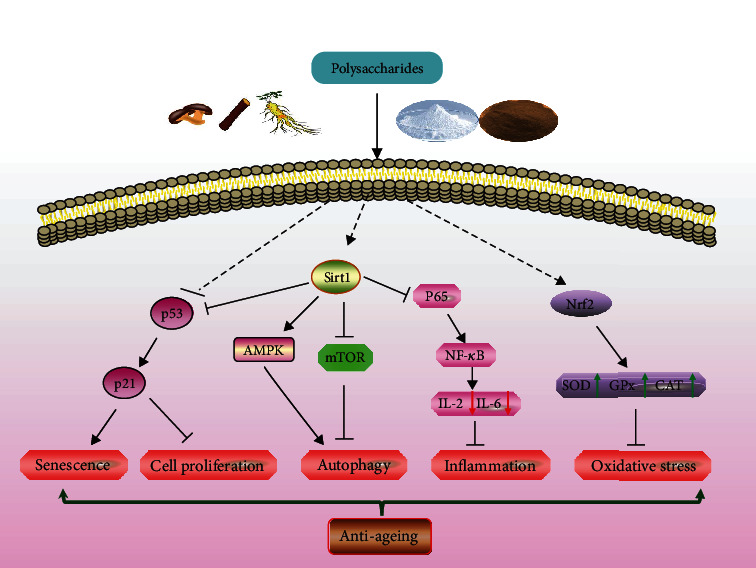
Antiaging mechanisms of polysaccharides and related signal pathways. AMPK: adenosine 5′-monophosphate-activated protein kinase; SOD: superoxide dismutase; GPx: glutathione peroxidase; CAT: catalase enzymes; mTOR: mammalian target of rapamycin; NF-*κ*B: nuclear factor kappa-B; Nrf2: nuclear factor E2-related factor 2.

The p53 signal pathway plays an important role in cellular senescence. The cellular senescence pathway can be triggered by inflammatory factors, leading to increased expression of the cellular oncogene p53, which in turn leads to the expression of its downstream target protein p21. The activation of p21 protein could block the progression of cells from G1 to the S phase, causing a transient cell cycle arrest and ultimately cellular senescence [115]. It was found that oral administration of DOPs reduced p53 expression and increased Bcl-2 expression in the ovaries of aging mice, thereby treating premature ovarian failure due to natural aging [116]. Injections of ASPs reduced the expression of p53 and p21 proteins in mice [59], and gavage of MPP reduced the expression of p21 and p53 proteins in the liver and brain of aging mice, thereby delaying cellular aging [64]. The above experiments suggest that polysaccharides regulate the p53 signal pathway to slow down aging. The relationship between natural product polysaccharides and aging-related signal pathways is listed in Table 2, and for the antiaging mechanisms of polysaccharides, see Figure 1.

## 6. Conclusions and Perspectives

Aging is a complex physiological process influenced by multiple factors, particularly environmental and genetic factors. The carbohydrate component of the human diet is derived almost exclusively from plant sources, while they also have other implications for diet and health. Recent studies indicate that polysaccharides play an important role in antiaging. More and more polysaccharides are used to develop antiaging functional foods. From the viewpoint of aging theory, antioxidant reagents usually have antiaging function and many polysaccharides also have strong antioxidant ability. Besides this, recent investigations show that many polysaccharides could target antiaging key molecules such as mTOR, AMPK, and p53, and the antiaging mechanism seems to be resolved at the gene and molecule levels. Up to now, whether dietary polysaccharides could be absorbed directly into the blood in the digestive tract is still controversial, and many researchers believe that polysaccharides cannot be absorbed directly into the blood, but there is also evidence that dietary polysaccharides could be detected in the blood, suggesting that at least some of them are directly absorbed by digestive gut, but the amount that could be detected in the blood is still very small; this suggests that it may not be the most important way for polysaccharides to play an antiaging role. With the development of investigations on intestinal microflora, more and more evidences show that polysaccharide could change intestinal microflora, and the change of intestinal microflora will inevitably lead to the change of its metabolic products, and these products could enter the blood and regulate AMPK, mTOR, and other aging-related gene expression in different cells. Therefore, it will be an important research direction to study the antiaging effect of polysaccharides through the change of intestinal microflora.

## Figures and Tables

**Table 1:** Antiaging effects of natural product polysaccharides.

Pharmacological action	Polysaccharides	Experiment model	Functions and mechanisms	Reference
Enhance vitality	*Porphyra yezoensis* polysaccharide (0.2%, 1% diets)	*Drosophila*	Delayed aging process, increased mating capacity	[31]
Antioxidation	Polysaccharide from *Cordyceps cicadae* (0.1%, 0.5%, and 1% diets)	*Drosophila*	Increased activities of CAT and GSH-Px, inhibited MDA formation, and upregulated the expression level of antioxidant-related genes: CAT, SOD1, and MTH	[32]
Antioxidation	Partially purified *Chlorella pyrenoidosa* polysaccharides (0.25%, 0.5%, and 1% diets)	*Drosophila*	Scavenged hydroxyl, 1,1-diphenyl-2-picrylhydrazyl, superoxide, and hydroxyl radicals. Increased the activity of endogenous antioxidant enzymes, superoxide dismutase, glutathione peroxidase, and catalase	[33]
Antioxidation	*Sargassum fusiforme* fucoidan (0.4 g/L, 0.8 g/L, and 1.6 g/L)	*Drosophila*	Improved activities of the antioxidant enzymes: SOD, CAT, and GSH-Px, and reduced the MDA and GSSG contents in older flies	[34]
Anti-inflammatory	*Premna microphylla* Turcz polysaccharide (0.25%, 0.5%, and 1% diets)	*Drosophila*	Reduced microvillus rupture in the midgut, restored the nuclear structure, and improved the expression levels of immune-related genes in inflamed *Drosophila*—especially defensin	[35]
Antioxidation	*Rehmannia glutinosa* polysaccharide (5 mg/mL)	*Caenorhabditis elegans*	Activated the antioxidant enzyme system under oxidative stress by promoting nuclear localization of DAF-16. Improved the activities of SOD and CAT, removed excess ROS, and reduced the expression of lipofuscin	[36]
Antioxidation	*Auricularia auricula* polysaccharide (0.1, 0.2, 0.4, and 0.8 mg/mL)	*Caenorhabditis elegans*	Improved the antioxidant defense system and upregulated the oxidative stress-related genes for stress damage prevention	[37]
Antioxidation	Polysaccharide from *Sophora moorcroftiana* seeds (2 mg/mL, 4 mg/mL)	*Caenorhabditis elegans*	Extended the life span and reproduction, increased the oxidative stress resistance and antimicrobial capacity	[19]
Antioxidation	*Polygonatum sibiricum* polysaccharide (100 mg/kg)	Rat	Increased the activities of SOD and GSH PX in kidney tissue, reduced the MDA content and *β*-galactosidase activity, regulated the Klotho-FGF23 endocrine axis, alleviated oxidative stress, and balanced calcium and phosphorus metabolisms	[39]
Antioxidation	Polysaccharide from *Athyrium multidentatum* (Doll.) Ching (100, 200, and 300 mg/kg)	Mouse	Attenuated D-galactose-induced oxidative stress and cell apoptosis by activating the PI3K/AKT pathway	[40]
Inhibition of granulosa cell apoptosis	*Fructus corni* polysaccharides (1.11 g/kg)	Mouse	Restored the estradiol and progesterone level, reduced the luteinizing hormone and follicle-stimulating hormone levels, increased Bcl-2, and reduced Bax and cleaved caspase-3	[112]
Antioxidation	*B. chinense* polysaccharides (0.8 and 1.6 mg/mL)	Mouse lung endothelial cells	Scavenged and resisted the H_2_O_2_-induced senescence	[43]
Antioxidation	*Angelica sinensis* (Oliv.) Diels (Apiaceae) polysaccharides (20 *μ*g/mL, 100 *μ*g/mL)	Endothelial progenitor cells (EPCs)	Augmented the Akt/hTERT phosphorylation and inhibited the oxidative stress	[44]
Inhibition of oxidative damage	*Angelica sinensis* polysaccharides (200 mg/kg)	Mouse hematopoietic stem cells	Decreased the percentage of SA-beta-Gal-positive cells, the ratio of G1 stages, and the production of ROS. Downregulated the expression levels of p16 mRNA	[45]
Antiapoptosis	*Lycium barbarum* polysaccharides (400 mg/L)	Human lens epithelial cells	Reduced the H_2_O_2_-induced cell apoptosis, ROS generation, mitochondrial membrane potential loss, and MDA levels and inhibited the H_2_O_2_-induced downregulation of Bcl-2. Upregulated Bax proteins and increased the SOD and GSH enzyme activities levels. Attenuated H_2_O_2_-induced cellular senescence	[46]
Antiapoptosis	*Lycium barbarum* polysaccharides (1.0, 2.0, 3.0, and 4.0 mg/mL)	Zebrafish embryos	Decreased the expression of aging-related genes such as p53, p21, and Bax. Increased Mdm2 and TERT genes	[47]
Improve cell viability	*Lycium barbarum* polysaccharides (100 mg/L)	HUVECs	Increased cell viability and decreased p53 and p16 expression levels	[48]
Increase the subjective feelings of general well-being	*Lycium barbarum* polysaccharides (120 mL of GoChi/day)	Healthy adults	Reduced fatigue and stress, improved the neurologic/psychologic performance and gastrointestinal functions	[49]
Antioxidation	*Lycium barbarum* polysaccharides (120 mL of GoChi/day)	Healthy adults	Increased the antioxidant efficacies by stimulating endogenous factors	[50]

Notes: CAT: catalase; FGF23: fibroblast growth factor 23; GSH-PX: glutathione peroxidase; GSSG: glutathione disulfide; MDA: malondialdehyde; MTH: methuselah; MDM2: mouse double minute 2 homolog; PI3K: phosphatidyl inositol 3-kinase; AKT: protein kinase B; ROS: reactive oxygen species; SOD: superoxide dismutase; hTERT: telomerase reverse transcriptase; GSSG: glutathione disulfide.

**Table 2:** Natural product polysaccharides and aging-related signal pathways.

Signal pathway	Polysaccharides	Experiment model	Functions and mechanisms	Reference
Sirt-1 regulation	*Lactobacillus plantarum* 69-2 combined with galacto-oligosaccharide (500 mg/kg)	Aging mouse model	Improved gut microbiota regulation, increased short-chain fatty acid levels, and activated the hepatic AMPK/SIRT1 regulatory pathway	[72]
	*Momordica charantia* polysaccharide (200 mg/kg)	Neural stem cells	The neural stem cell neuronal differentiation promoted because of the deacetylated *β*-catenin by SIRT1	([73]; [74])
	*Lycium barbarum* polysaccharide (100, 200, and 400 mg/L)	Human lens epithelial cell line SRA01/04 cells	Upregulated Sirt1 and Bcl-2, suppressed cell death related genes	[76]
	*Lycium barbarum* polysaccharide (100 mg/kg)	Diabetic rats	Increased cell proliferation, inhibited cell apoptosis, and regulated SIRT1/HIF-1*α* expression	[78]
	*Tremella* polysaccharides (10 *μ*g/mL)	Human epithelial A549 lung cancer cells	Activated SIRT1 and inhibited the LPS-induced ROS production, apoptosis, and autophagy	[79]
	*Astragalus* polysaccharide (100 mg/kg)	BALB/c male mice	Restored imbalance between mitochondrial fusion-fission processes, activated mitophagy, decreased PGC-1*α* expression, and ameliorated mitochondrial dysfunction	[80]
	*Astragalus* polysaccharide (700 mg/kg)	Male Sprague-Dawley rats	Suppressed abnormal glycolipid metabolism and insulin resistance by improving hepatic SIRT1-PPAR*α*-FGF21 intracellular signaling. Reduced chronic inflammation by attenuating hepatic steatosis	[81]
	*Astragalus* polysaccharide (2.5, 25, and 50 *μ*g/ml)	Retinal pigment epithelial cells	Inhibited ER stress and subsequent apoptosis via regulating miR-204/SIRT1 axis	[82]
	Okra polysaccharides (200 or 400 mg/kg)	Diabetic mice	Suppressed apoptosis and oxidative stress through activating the AMPK-Sirt1-PGC-1*α* signaling axis	[83]
	*Tremella fuciformis* polysaccharide (100, 200, and 300 *μ*g/mL)	Human skin fibroblasts	Alleviated hydrogen peroxide-induced oxidative stress and apoptosis	[84]
	*Apios americana* Medikus tuber polysaccharide (30 mg/mL)	RAW 264.7 cells	Suppressed NO release, inflammatory cytokines, oxidative stress, and mitochondrial dysfunction	[85]
mTOR regulation	*Ganoderma lucidum* polysaccharide (1.2 mg/mL)	Intestinal porcine epithelial cell line	Inhibited cell apoptosis and autophagy through the promotion of Akt phosphorylation and mammalian target of rapamycin (mTOR)	[95]
	*Astragalus* polysaccharide (50, 100, and 200 *μ*g/mL)	Fibroblast-like synoviocytes	Inhibited cell growth and proinflammatory response by enhancement of autophagy via PI3K/AKT/mTOR inhibition	[96]
	A sulfated glucan from *Antrodia cinnamomea* (650 *μ*g/mL)	Lung cancer cells	Reduced lung cancer cell viability via inhibition of the EGFR and mTOR activities	[97]
	Pectic bee pollen polysaccharide from *Rosa rugosa* (0.1 mg/mL)	Obese mice	Alleviated diet-induced hepatic steatosis and insulin resistance via AMPK/mTOR-mediated autophagy	[98]
	Fucoidan from seaweed *Fucus vesiculosus* (50, 100, and 200 *μ*g/mL)	A549 lung cancer cells	Exhibited antimetastatic effect on A549 lung cancer cells via the downregulation of ERK1/2 and Akt-mTOR as well as NF-*κ*B signaling pathways	[99]
	*Pleurotus nebrodensis* polysaccharide (200 *μ*g/mL)	A549 tumor-bearing mice	Activated AMPK phosphorylation, inhibited PI3K/AKT phosphorylation, suppressed the activation of the mTOR signaling pathway, and decreased the expression of the translation-related protein P70S6K	[100]
	Chitosan oligosaccharide (500 mg/kg)	Mouse model of colitis-associated colorectal cancer	Suppressed tumor progression through AMPK activation and suppression of NF-kappaB and mTOR signaling	[101]
AMPK regulation	*Astragalus* polysaccharide (10 *μ*g/mL)	Mouse 3T3-L1 preadipocytes	Improved insulin sensitivity via AMPK activation	[19]
	*Astragalus* polysaccharide (700 mg/kg)	Type 2 diabetes mellitus rat model	Alleviated glucose toxicity by increasing liver glycogen synthesis and skeletal muscle glucose translocation via AMPK activation	[105]
	*Astragalus* polysaccharide (200 mg/kg)	Porcine alveolar macrophages	Attenuated ochratoxin A-induced immune stress by activating the AMPK/SIRT-1 signaling pathway	[106]
	*Astragalus* polysaccharide (400 *μ*g/mL)	RAW264.7 cells	Ameliorated palmitate-induced proinflammatory responses through AMPK activation	[107]
	Polysaccharide from *Fuzi* (200 mg/kg)	H9c2 cells	Increased autophagy through AMPK/mTOR pathway activation	[108]
	*Schisandra chinensis* acidic polysaccharide (10, 20, and 40 mg/kg)	Mouse model of acute liver injury	Diminished MDA levels, GSH, and cleaved caspase-3 expression, elevated the expression of p-AMPK, p-Akt, and p-glycogen synthase kinase 3*β*, and partially reversed acetaminophen-induced liver injury	[110]
	*Irpex lacteus* polysaccharide-enriched extract (0.04, 0.2, and 1.0 g/kg)	Mouse	Enhanced the endurance capacity of mouse by elevating antioxidant associated with the AMPK pathway	[41]
	Low molecular weight fucoidan (40 and 80 mg/kg)	Obese diabetic db/db mice	Prevented NAFLD by activating the SIRT1/AMPK/PGC1*α* signaling pathway	[113]
	Chicory polysaccharides (100 and 200 mg/kg)	High-fat diet rats	Attenuated high-fat diet induced nonalcoholic fatty liver disease via AMPK activation	[114]
p53 regulation	Polysaccharides from *Dendrobium officinal* (70 mg/kg)	Female mice	Alleviated damage caused by aging through the inhibition of the nuclear NF-*κ*B and p53/Bcl-2-mediated signaling pathways	[116]
	*Angelica* polysaccharide (140 mg/kg)	Nestin-GFP transgenic mouse brain tissues and neural stem cells	Delayed aging speed by protecting neural stem cells and upregulating the p53/p21 signaling pathway	[59]
	*Yulangsan* polysaccharide (0.6 g/kg)	D-Galactose-treated mice	Suppressed the aging process by decreasing p21 and p53 gene expressions in the liver and brain	[64]

Notes: AMPK: adenosine monophosphate-activated protein kinase; ERK: extracellular signal-regulated kinase; FGF21: fibroblast growth factor 21; PPAR: peroxisome proliferator-activated receptor; PI3K: phosphatidyl inositol 3-kinase; AKT: protein kinase B; ROS: reactive oxygen species; SIRT: sirtuin; mTOR: the mammalian target of rapamycin; NAFLD: nonalcoholic fatty liver disease; LPS: lipopolysaccharides; NO: nitric oxide; AMPK: adenosine monophosphate-activated protein kinase; PGC: primordial germ cell; MDA: malondialdehyde; GSH-PX: glutathione peroxidase; HIF: hypoxia-inducible factor.

## Abbreviations

AMPK: Adenosine monophosphate-activated protein kinase
ASP: *Angelica sinensis* polysaccharides
APS: *Astragalus* polysaccharide
AMC: *Athyrium multidentatum* (Doll.) Ching
BCPs: *Bupleurum chinense* polysaccharides
*C. elegans*: *Caenorhabditis elegans*
CAT: Catalase
CPs: Chicory polysaccharides
PCPPs: *Chlorella pyrenoidosa*
CP: *Cordyceps cicadae*
CS: *Cordyceps sinensis*
CSP: *Cynomorium songaricum* polysaccharide
DOP: *Dendrobium officinale* polysaccharides
ERK: Extracellular signal-regulated kinase
FGF21: Fibroblast growth factor 21
PFC: *Fructus corni*
PGC: Primordial germ cell
GOS: Galacto-oligosaccharide
GLPs: *Ganoderma lucidum* polysaccharides
GSH-PX: Glutathione peroxidase
GSSG: Glutathione disulfide
eGFR: Estimated glomerular filtration rate
HIF: Hypoxia-inducible factor
LEN: Lentinan
LMWF: Low molecular weight rockweed polysaccharide
LBP: *Lycium barbarum* polysaccharides
MDA: Malondialdehyde
MPP: *Millettia pulchra* polysaccharide
MTH: Methuselah
MAPK: Mitogen-activated protein kinases
MDM2: Mouse double minute 2 homolog
NO: Nitric oxide
NAFLD: Nonalcoholic fatty liver disease
RBPP-P: Pectic bee pollen polysaccharide
POS: Pectic oligosaccharides
PPAR: Peroxisome proliferator-activated receptor
PI3K: Phosphatidyl inositol 3-kinase
POPs: *Pleurotus ostreatus* polysaccharides
PS: *Polygonatum sibiricum*
pPMTLs: *Premna microphylla* Turcz
AKT: Protein kinase B
ROS: Reactive oxygen species
SFPS: *Sargassum fusiforme* polysaccharides
SCPs: *Schisandra chinensis* polysaccharides
Sirt: Sirtuin
SOD: Superoxide dismutase
hTERT: Telomerase reverse transcriptase
T-AOC: Total antioxidant capacity
mTOR: The mammalian target of rapamycin
TFPs: *Tremella fuciformis* polysaccharides.
